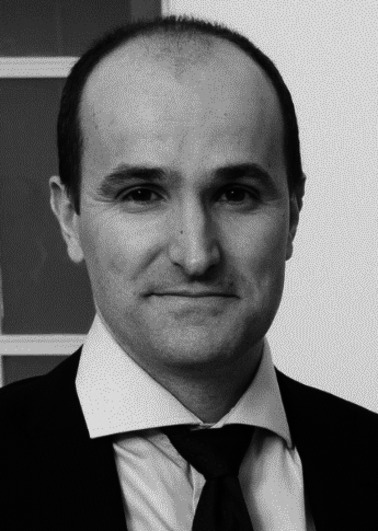# Miniaturized Chemical Tags for Optical Imaging

**DOI:** 10.1002/anie.202204788

**Published:** 2022-07-21

**Authors:** Sam Benson, Fabio de Moliner, William Tipping, Marc Vendrell

**Affiliations:** ^1^ Centre for Inflammation Research The University of Edinburgh Edinburgh EH16 4TJ UK; ^2^ Centre for Molecular Nanometrology The University of Strathclyde Glasgow G1 1RD UK

**Keywords:** Bioconjugates, Fluorophores, Probes, Raman Spectroscopy

## Abstract

Recent advances in optical bioimaging have prompted the need for minimal chemical reporters that can retain the molecular recognition properties and activity profiles of biomolecules. As a result, several methodologies to reduce the size of fluorescent and Raman labels to a few atoms (e.g., single aryl fluorophores, Raman‐active triple bonds and isotopes) and embed them into building blocks (e.g., amino acids, nucleobases, sugars) to construct native‐like supramolecular structures have been described. The integration of small optical reporters into biomolecules has also led to smart molecular entities that were previously inaccessible in an expedite manner. In this article, we review recent chemical approaches to synthesize miniaturized optical tags as well as some of their multiple applications in biological imaging.

## Graphical abstract

This Minireview covers some of the recent advances in the development of small chemical tags for optical bioimaging, from their chemical synthesis and characterization to their application in different biological systems, such as cells, tissues and whole organisms. It focuses on the design of fluorescent and Raman labels and features examples of metabolites, amino acids, nucleic acids as well as organelle and cell‐targeting probes.

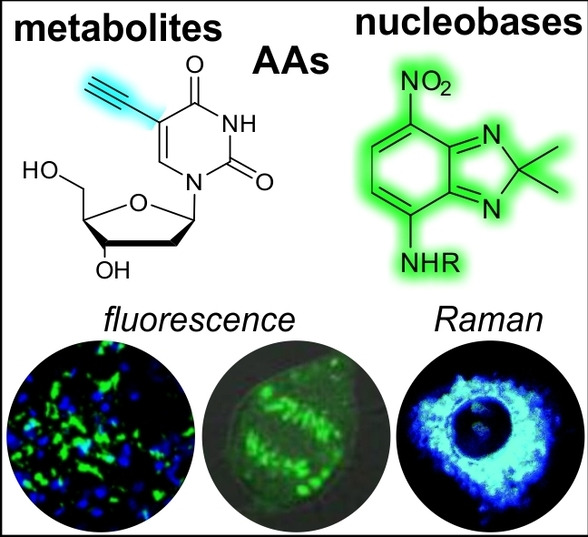

## 1 Introduction

Molecular imaging has transformed the way researchers analyze biological systems, providing a means to study how molecules and cells function in real time. Among different modalities, optical imaging features multiplexing capabilities, low cost, and the possibility to directly acquire molecular information from cells, tissues and organisms with high spatial and temporal resolution.^[1]^ These properties have been exploited by chemists to synthesize imaging probes related to numerous biological processes, from signaling pathways to cell‐to‐cell interactions. However, one of the main challenges synthetic chemists face when designing probes is the potential interference of the optical labels (e.g., fluorophores, vibrational groups) in the biological activity and recognition properties of their molecular targets.

**Figure 1:**
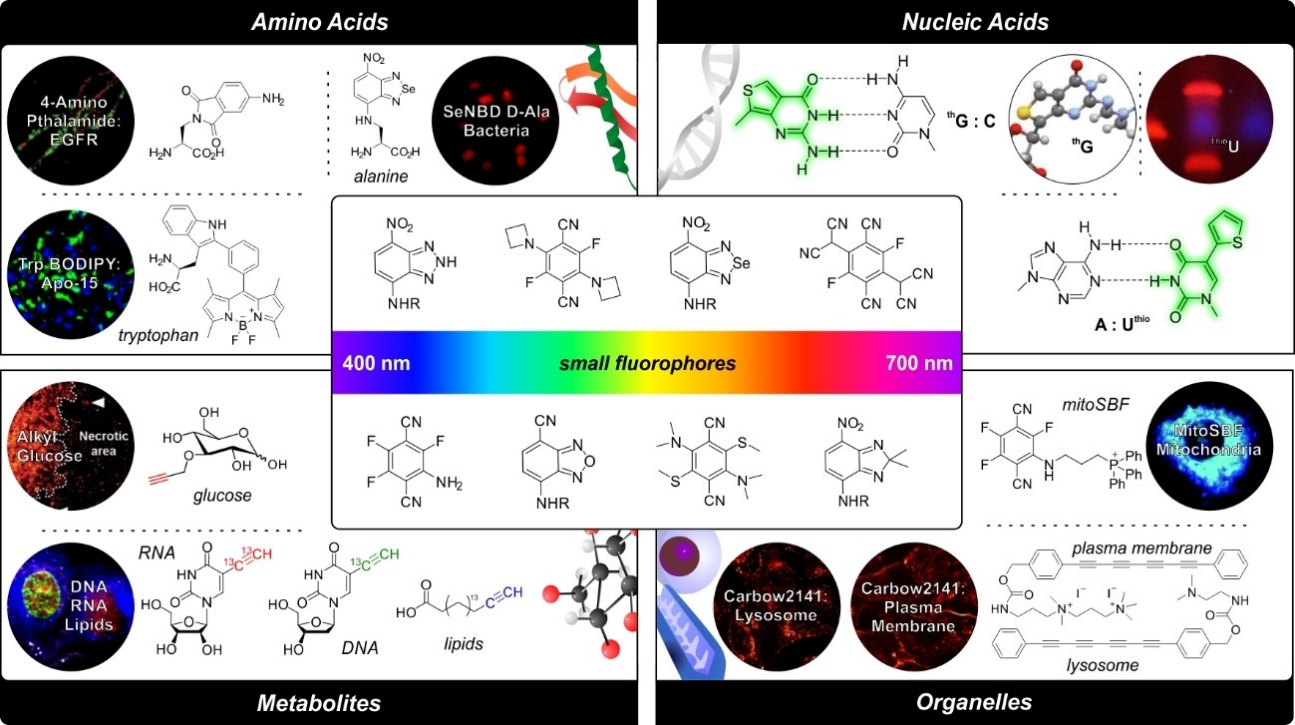
Miniaturized chemical labels for optical imaging. Representative chemical structures of miniaturized tags for imaging four major targets, namely amino acids, nucleic acids, metabolites and subcellular organelles. Examples include fluorescent amino acids and nucleobases as well as Raman‐active molecules for metabolite and organelle imaging. Inset: Representative chemical structures of small fluorophores with emission in the UV/Visible spectrum. Copyrights (2011–2021) from American Chemical Society, Wiley‐VCH, The Royal Society of Chemistry and Springer Nature under a creative commons license.

In the recent years, multiple chemical approaches have been devised to miniaturize fluorescent and Raman‐active tags for minimally invasive labeling of biomolecules while retaining sensitivity and compatibility with fluorescence and Raman microscopy (Figure 1).^[2]^ In the design of fluorescent probes, the range of chemical strategies is broad and covers small sized and neutral fluorophores with tunable emission and non‐perturbative properties to fluorophores that can behave as surrogates of native building blocks (e.g., nucleobases, amino acids) for the preparation of fluorescent nucleic acids, peptides and proteins.^[3]^ With regards to Raman probes, most efforts have focused on the exploration of even smaller chemical groups (e.g., from triple bonds to deuterium atoms) with vibrational frequencies within the cell silent region of the Raman spectrum, where the limited sensitivity of Raman readouts can be overcome.

This Minireview article summarizes recent advances in chemical methodologies to reduce the size of optical labels to a few atoms (e.g., aryl‐based fluorophores, isotopes) and accelerate the construction of native‐like supramolecular structures. In addition to the design and synthesis of minimal chemical reporters, we will also review examples of how they can be integrated into metabolites and biomolecules to prepare smart molecular entities (e.g., fluorogenic peptides, ratiometric probes) that were previously inaccessible (Figure 1).

## 2 Small Environmentally Sensitive Fluorophores

**Figure 2:**
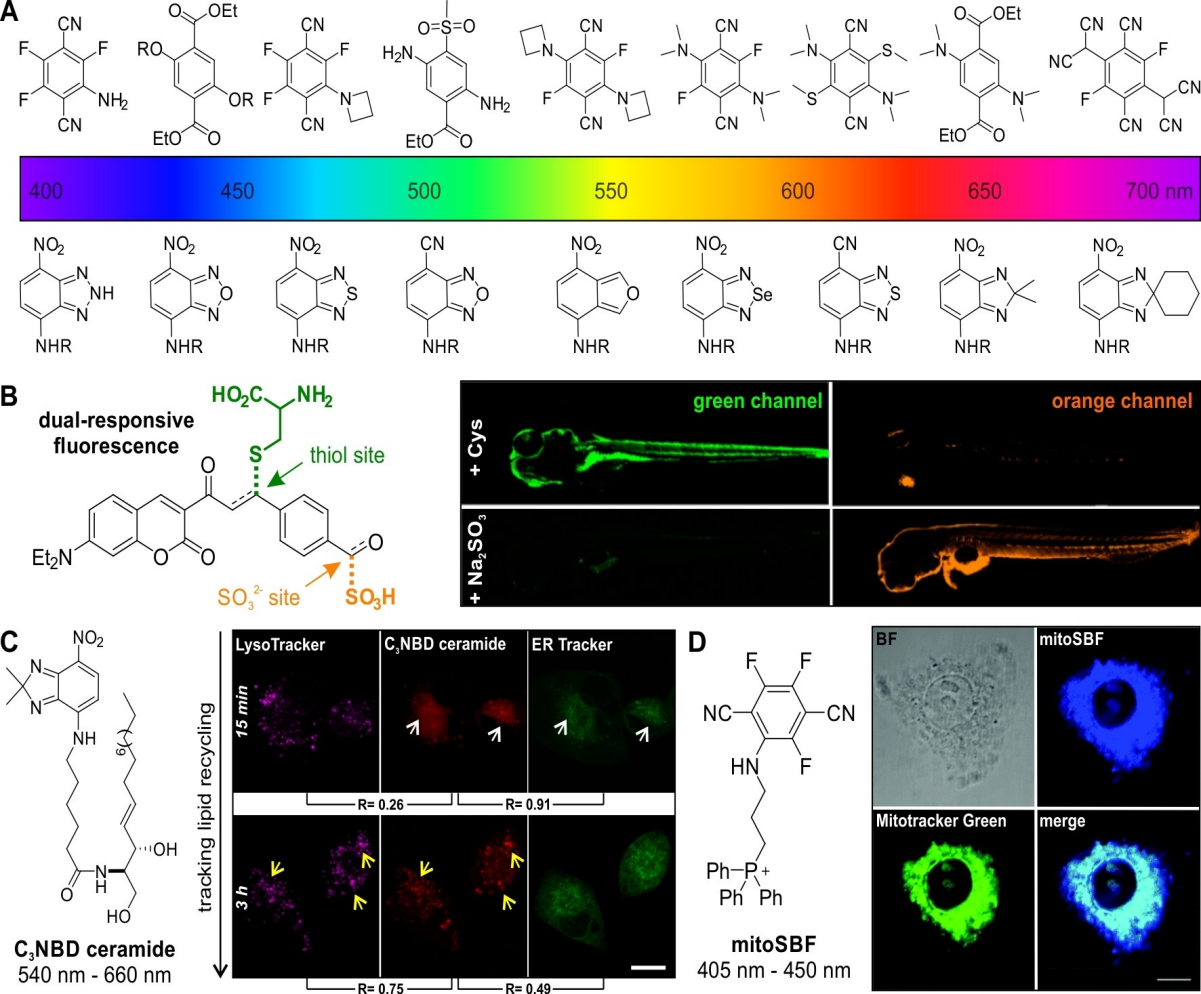
Small and tunable organic fluorophores. A) Structures of single benzene carbocycle (top) and benzodiazole (bottom) fluorophores spanning blue to NIR regions of the visible spectrum. B) Dual‐site coumarin probe for the simultaneous detection of cysteine and its metabolite SO_2_ and confocal microscopy fluorescence images of zebrafish upon incubation with the probe. Reproduced with permission.^[9d]^ Copyright 2017 American Chemical Society. C) Structure of C_3_‐NBD ceramide and confocal microscopy fluorescence images of A549 cells upon incubation with the probe (red), LysoTracker (magenta) and ER (endoplasmic reticulum) Tracker Green (green). Reproduced with permission.^[15a]^ Copyright 2019 Wiley‐VCH. D) Chemical structure of the mitochondria‐targeting probe mitoSBF and colocalization with Mitotracker Green (green) in HepG2 cells. Reproduced with permission.^[23]^ Copyright 2019 The Royal Society of Chemistry.

Fluorescent molecules have been extensively researched over the last decades and revolutionized the way we investigate biological processes in vitro and in vivo. While the chemical structures of fluorophores can vary widely,^[4]^ many of them (e.g., fluoresceins, rhodamines, cyanines) are charged structures with larger sizes than those of messenger molecules found in cells. Because such chemical properties can affect the intracellular localization and bioactivity profiles of metabolites,^[5]^ their application in bioconjugate chemistry often requires the introduction of suitable spacers.^[6]^ Alternatively, smaller fluorophores (≈200–300 Da) with simpler chemical structures can facilitate the minimally disruptive labeling of biomolecules (Figure 2).

The oldest example of small sized fluorophores are coumarins, which were first synthesized by Perkin in 1868.^[7]^ Since they possess excellent biocompatibility and bright fluorescence emission, they have been employed as chemosensors for multiple analytes.^[8]^ For instance, activatable coumarin probes have been reported for the detection of biothiols (e.g., cysteine, glutathione, homocysteine), exploiting Michael‐type nucleophilic additions as the fluorescence trigger (Figure 2).^[9]^ More recently, the replacement of the oxygen atom in the coumarin scaffold with a difluoromethylene unit led to small fluorophores with emission wavelengths in the near infrared (NIR) range. The group of Schnermann re‐designed the heterocyclic coumarin core and synthesized a palette of small and NIR‐emitting Fluoro‐Coumarin dyes with emission maxima wavelengths up to 800 nm.^[10]^ These dyes displayed strong emission in hydrophobic environments, which was exploited for targeting lipid droplets in cell imaging experiments.

Nitrobenzoxadiazoles (NBD) are another family of small sized fluorophores commonly used for biological imaging. Building from the original NBD chloride, N‐substituted NBD amines have been described both as environmentally sensitive dyes and as turn‐on or turn‐off probes.^[11]^ The NBD scaffold is highly versatile, and the optical properties can be fine‐tuned by making changes in its chemical structure. For instance, VanVeller and co‐workers reported the replacement of the electron‐withdrawing nitro groups with cyano groups to obtain derivatives with red‐shifted emission wavelengths and higher sensitivity to polarity changes.^[12]^ The same team prepared nitroisobenzofuran structures showing a ≈100 nm bathochromic shift in emission compared to analogous scaffolds based on benzofurazan, albeit showing lower quantum yields.^[13]^ Furthermore, the single replacement of bridging heteroatoms in benzoxadiazoles has been reported to modify their spectral properties.^[14]^ Our group described SCOTfluors as a family of small fluorophores with variable bridging units and emission wavelengths covering from the blue to the NIR regions of the visible spectrum.^[15]^ This chemical platform enabled multicolor labeling and live cell tracking of metabolites (e.g., glucose, lactate, ceramides) that retained affinity for their cognate transporters, including some of the smallest NIR fluorophores reported to date (Figure 2).^[16]^

Another emerging class of small sized fluorophores are single benzene ring carbocycles. Fully carbocyclic fluorescent molecules such as aminonaphthalenes and azulene had long been known,^[17]^ but only recently reported as fluorogenic bioorthogonal probes for intracellular imaging of organelles,^[18]^ labeling of β‐lactam antibiotics^[19]^ and two‐photon fluorescent probes for imaging reactive oxygen species in cells and tissues.^[20]^ Other approaches to design tunable and miniaturized fluorophores have exploited the functionalization of the simplest aromatic system (i.e., benzene) with variable patterns of electron‐donating and electron‐withdrawing substituents to generate chemically diverse push‐pull systems. For instance, the group of Fang reported solvatochromic fluorophores by simple variation of the number and the position of benzoate and azetidine moieties.^[21]^ Similar strategies have also been exploited for other applications, from the preparation of red emissive organic crystals to biomaterials for cell imaging and antibiotic evaluation.^[22]^ Of particular interest in this area, Yuan and co‐workers recently described SB‐Fluors as single benzene terephthalonitrile‐based structures covering the entire visible spectrum and having suitable properties for live cell imaging of mitochondria and quantification of HClO (Figure 2).^[23]^ Furthermore, compact bicylic triazapentalenes (TAP) have also been exploited as tunable fluorophores for biological imaging. These structures were first described by Namba and co‐workers, and several examples of functionalized TAPs have been reported for the detection of endogenous proteins and drugs as well as for the morphological analysis of cells during differentiation processes.^[24]^ Altogether, the design of novel and diverse minimally sized fluorophores is an extremely active research topic. The development of fluorescent frameworks going beyond the conventional extended planar polycycles represents a promising avenue to fine‐tune the optical and biological properties of small organic dyes.

## 3 A Fluorescent Alphabet of Nucleobases

**Figure 3:**
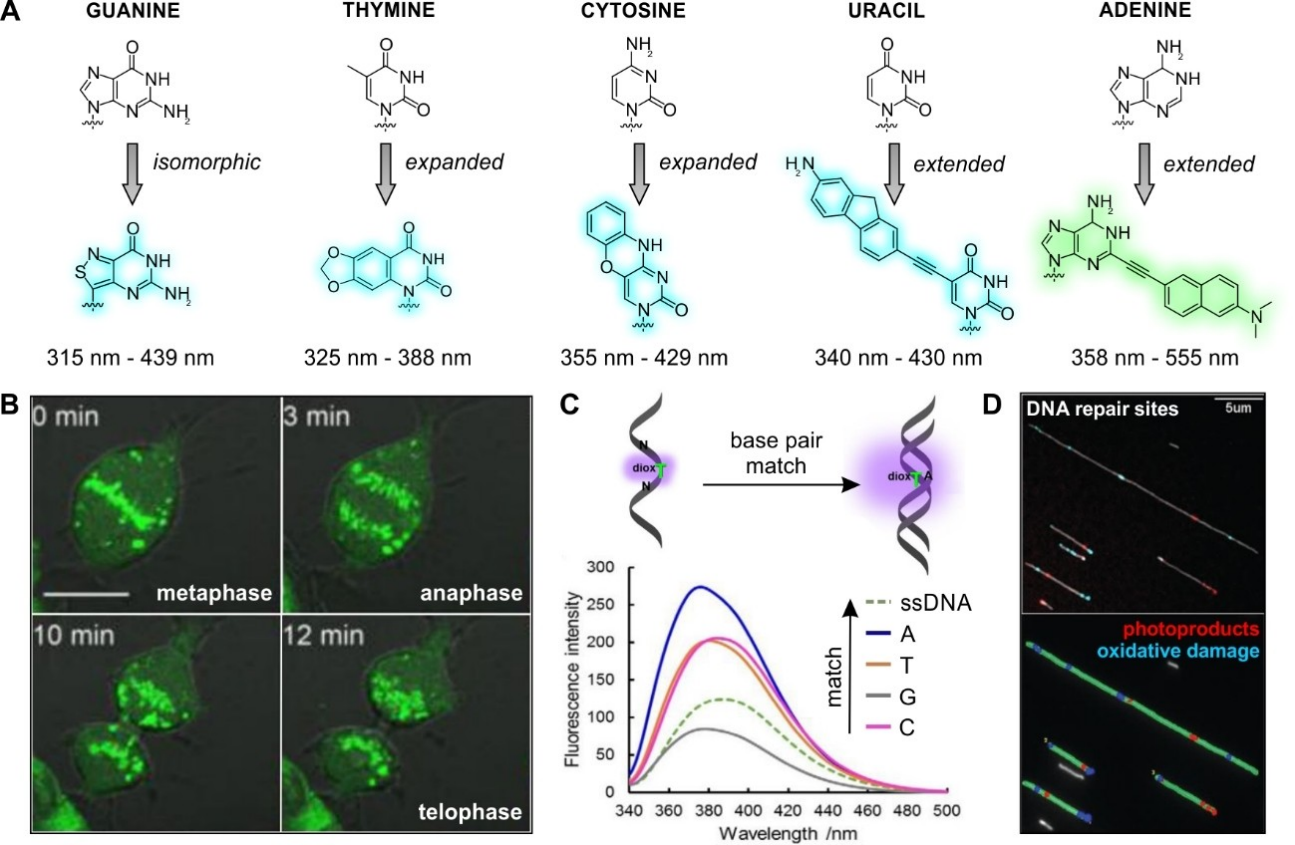
Miniaturized fluorescent nucleobases. A) Chemical structures of native nucleobases and representative analogues with their corresponding absorbance–emission wavelengths. B) Time‐lapse imaging of mitosis in U2‐OS cells after incorporation of the dCmBdp nucleotide. Scale bar: 20 μm. Adapted with permission.^[33a]^ Copyright 2018 American Chemical Society. C) Fluorescence spectra of single‐stranded DNA (ssDNA) and double‐stranded DNA (dsDNA) containing the nucleobase dioxT flanked by the bases AT (excitation: 330 nm). Adapted with permission.^[30]^ Copyright 2019 Wiley‐VCH. D) Simultaneous detection of photoproducts and oxidative DNA damage in HEK (human embryonic kidney) cells exposed to UVC radiation. Top panel: Fluorescence image of DNA labeled with the multilabeling reaction. Bottom panel: Image analysis where green color features DNA molecules, red highlights the photoproduct labels, and blue corresponds to oxidative damage sites. Adapted with permission.^[37]^ Copyright 2019 The Royal Society of Chemistry.

Nucleic acids are key biomolecules to define cell function. Due to their complex interactions, fluorescent nucleic acids (FNAs) must have minimal interference in the molecular recognition properties of the native counterparts. Nucleobases have been derivatized to produce collections of fluorescent analogues (Figure 3),^[25]^ including the systematic modification of purines and pyrimidines to ensure compatibility with all pairings^[26]^ as well as adaptation to prepare single and double‐stranded nucleic acids.^[27]^

The derivatization of fluorescent nucleobases can be grouped into three types of molecular architectures: isomorphic, expanded and extended. Approaches that modify the heterocyclic cores of nucleobases are referred to as isomorphic. One of the most prominent isomorphic nucleobases is thienoguanosine, where the imidazole ring of guanine is replaced by a thiophene.^[28]^ More recent modifications include analogues where the thiophene ring was substituted by an isothiazole group to enhance enzyme recognition.^[29]^ Expanded analogues maintain the core structure of nucleobases while incorporating additional rings to improve their fluorescence properties. These analogues can be obtained by appending benzyl moieties onto the non‐base pairing region of the nucleobase[^25c^, ^30^] or by incorporating base pairing functional groups directly into new aromatic functionalities.^[31]^ Finally, extended architectures are the most common unnatural nucleobases, with the distal face being adaptable to modification on unsubstituted carbons. The position 5 in pyrimidines has been a prime focus of extended nucleobases because it is not directly involved in base pairing. Chemical modifications have been performed using Suzuki–Miyaura couplings to introduce aromatic moieties, such as benzofurans and naphthalenes.^[32]^ Alternatively, Sonogashira cross couplings enabled the introduction of small fluorophores to this position in pyrimidines^[33]^ and also in purines at different positions.^[34]^

The most widespread application of FNAs is their use for optical imaging, including the direct visualization of intracellular processes in live cells (e.g., mitosis as shown in Figure 3).^[33a]^ Fluorogenic nucleic acids have also enabled the identification of pairing mismatches, including probes where fluorophores switch their emission on or off in the presence or absence of complementary base pairs (Figure 3).[^25b^, ^27a^, ^30^] The group of Purse recently reported electron‐rich FNAs that exhibited turn‐on fluorescence when inserted into double‐stranded deoxyribonucleic acid (DNA) whereas electron poor FNAs showed decreased fluorescence intensities.^[35]^ Saito and co‐workers employed turn‐on fluorophores to identify abasic sites by monitoring specific optical features, such as fluorescence intensity or bathochromic shifts in emission wavelengths.^[34c]^ Furthermore, in combination with enzymatic removal of damaged bases, it has been possible to introduce fluorescent nucleobases into damaged sites of nucleic acids to identify specific regions and the extent of damage.^[36]^ Notably, this approach can be multiplexed to distinguish between oxidative and UV damage with different fluorophores (Figure 3).^[37]^ This concept has been recently adapted by the group of Kool to develop a miniaturized adenine nucleobase to identify the extent of DNA damage repair.^[25a]^ Briefly, the MUTYH protein excises undamaged adenine opposite oxidatively damaged guanine. The authors developed a turn‐on adenine analogue that fluoresces upon excision from the DNA duplex by MUTYH, thus allowing in vitro evaluation of small molecule MUTYH modulators. In summary, miniaturized fluorescent nucleobases provide the means to not only track their position within nucleic acids but also to study the interactions between polynucleotides and other biomolecules.

## 4 Fluorescent Amino Acids for Site‐Specific Peptide and Protein Tagging

Peptides and proteins play key roles in multiple biological processes; however, in their native form they do not emit bright fluorescence and chemical tags are needed to aid their detection. Several groups have demonstrated that coupling different fluorophores to similar peptide sequences can result in conjugates with different biomolecular properties (e.g., enzyme reactivity, intracellular localization),[^5^, ^38^] highlighting the need for non‐perturbative labeling strategies. Unlike fluorescent fusion proteins and self‐labeling tags ‐which involve large structural modifications‐, fluorescent amino acids are miniaturized alternatives to diversify peptides and proteins in a less disrupting manner.^[39]^

**Figure 4:**
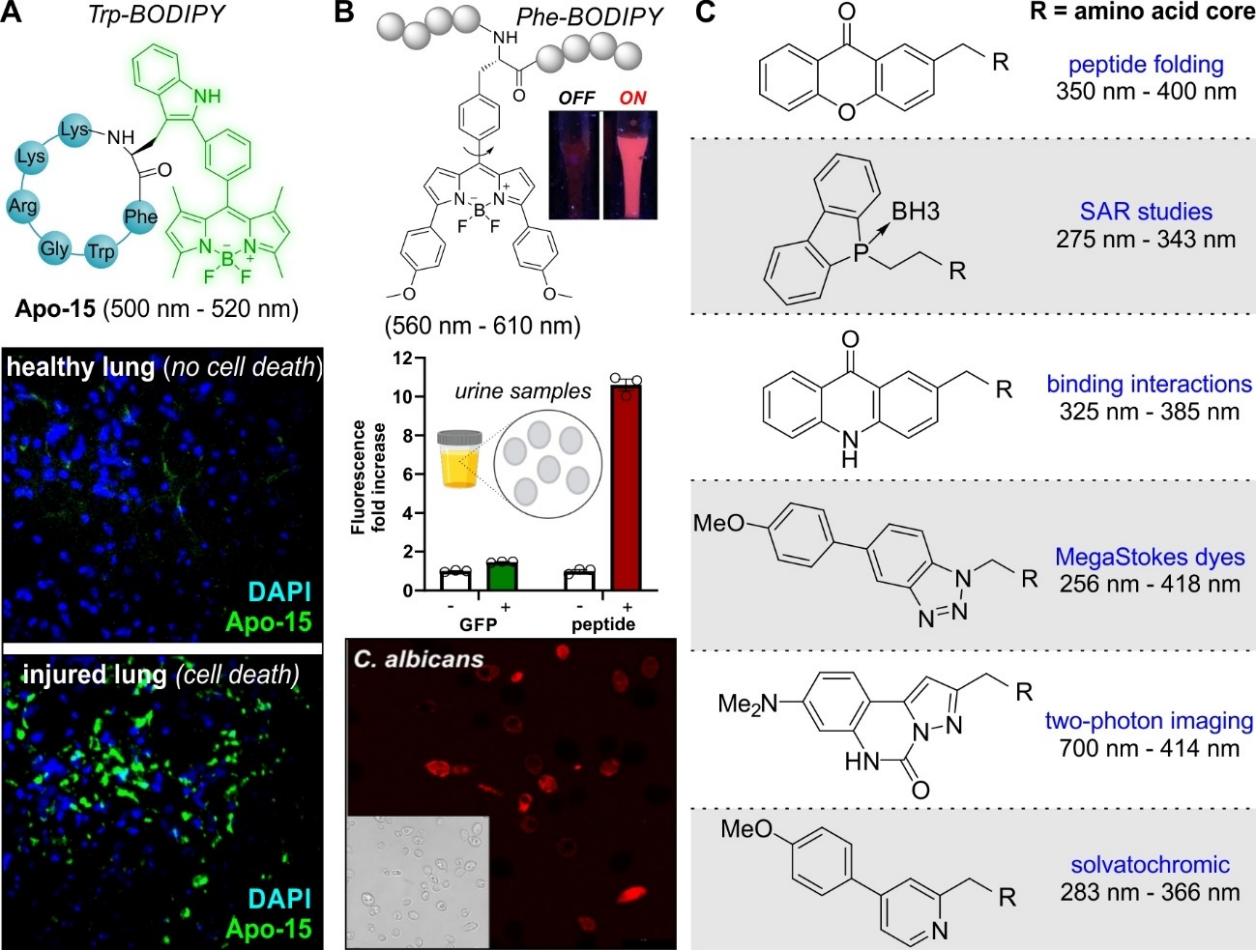
Small fluorescent amino acids for site‐specific peptide labeling. A) Chemical structure of the Trp‐BODIPY‐containing fluorogenic peptide Apo‐15 for in vivo imaging of apoptotic cells (green) in mouse lungs using intravital fluorescence microscopy. Adapted with permission.^[43]^ Copyright 2020 Springer Nature under a creative commons license. B) Antimicrobial peptides including red emitting Phe‐BODIPY amino acids as fluorogenic probes for the detection of fungal pathogens in human urine. Comparative detection of GFP‐labeled *Candida albicans* in urine samples using the GFP (green) and fluorogenic peptide (red) readouts. Confocal fluorescence microscopy of peptide‐stained *Candida albicans* cells. Adapted with permission.^[47]^ Copyright 2022 Wiley‐VCH. C) Representative examples of fluorescent amino acids with featured applications and absorbance‐emission wavelengths.

Our group developed Trp‐BODIPY as the first BODIPY‐based fluorogenic amino acid to be used as a Trp surrogate in antimicrobial peptides for imaging fungal pathogens in ex vivo human lungs.^[40]^ The synthesis of Trp‐BODIPY is straightforward, involving 4 steps with one key C−H activation reaction to directly couple tryptophan to an iodinated BODIPY scaffold without the need of any spacer.^[41]^ The scalability of Trp‐BODIPY has enabled its application for multiple peptide sequences,^[42]^ including the fluorogenic peptide Apo‐15 for imaging apoptotic cells in vivo (Figure 4).^[43]^ Subsequent efforts have led to other Trp‐based amino acids with 1) enhanced environmental sensitivity to monitor membrane fluidity in T cells^[44]^ or 2) longer emission wavelengths, such as the red fluorescent Trp(redBODIPY),^[45]^ which has been successfully adapted for solid‐phase peptide synthesis.^[46]^ Some of the latest examples in BODIPY amino acids have rendered smaller Phe‐based compounds (Figure 4), which retain the fluorogenic character of Trp‐BODIPY while reducing the overall size of the fluorophore,^[47]^ and Cys‐reactive activatable probes for the derivatization of small proteins.^[48]^

Trp‐based amino acids including smaller chemical modifications to boost their optical properties have been reported. Although they are typically not as bright as BODIPY‐containing amino acids, they still display enhanced optical properties when compared to the natural Trp. Some of these amino acids include azatryptophans (emission ≈400–420 nm)^[49]^ and cyanotryptophans (emission ≈415 nm),^[50]^ which have been used in the preparation of structurally modified proteins as well as tricyclic tryptophan derivatives with large Stokes shifts (≈150–160 nm).^[51]^

Alternative scaffolds have been successfully employed to prepare fluorescent amino acids with suitable properties for in vitro biomolecular and bioimaging studies (Figure 4). These include xanthone‐based amino acids for studying peptide folding by triplet–triplet energy transfer,^[52]^ acridon‐2‐ylalanines for lifetime and Förster resonance energy transfer studies,^[53]^ conformationally rigid pyrazoloquinazoline α‐amino acids for two‐photon NIR excitation,^[54]^ phospholyl and benzotriazoles amino acids with large Stokes shifts (>160 nm),^[55]^ and solvatochromic amino acids containing β‐pyridyl^[56]^ or phthalimide groups for the preparation of environmentally sensitive peptides retaining bioactivity (e.g., transmembrane fragments of the epidermal growth factor receptor).^[57]^

The miniaturized size of fluorescent amino acids has also been exploited to utilise the machinery of living cells for site‐specific labeling of proteins under physiological conditions. Examples of these structures include the seminal work by the groups of Schultz, Chin and others on genetically encoded amino acids (e.g., CouA, ANAP),^[58]^ which have been reviewed in detail elsewhere.^[39]^ More recently, D‐fluorescent amino acids have been developed for in situ labeling of bacterial cell walls, including multiplexable tools for multicolor imaging of peptidoglycan biosynthesis in bacteria^[59]^ as well as molecular rotors for real‐time imaging and high‐throughput assays.^[60]^ Altogether, the repertoire of small fluorescent amino acids with broad diversification of chemical structures (e.g., Phe and Trp‐based), optical readouts (e.g., variable emission wavelengths, long Stokes shifts, environmental sensitivity) and biological properties (e.g., genetically encodable) will accelerate the design of new artificial proteins and bioactive peptides for optical imaging.

## 5 Miniaturized Raman Labels for Metabolic Imaging

**Figure 5:**
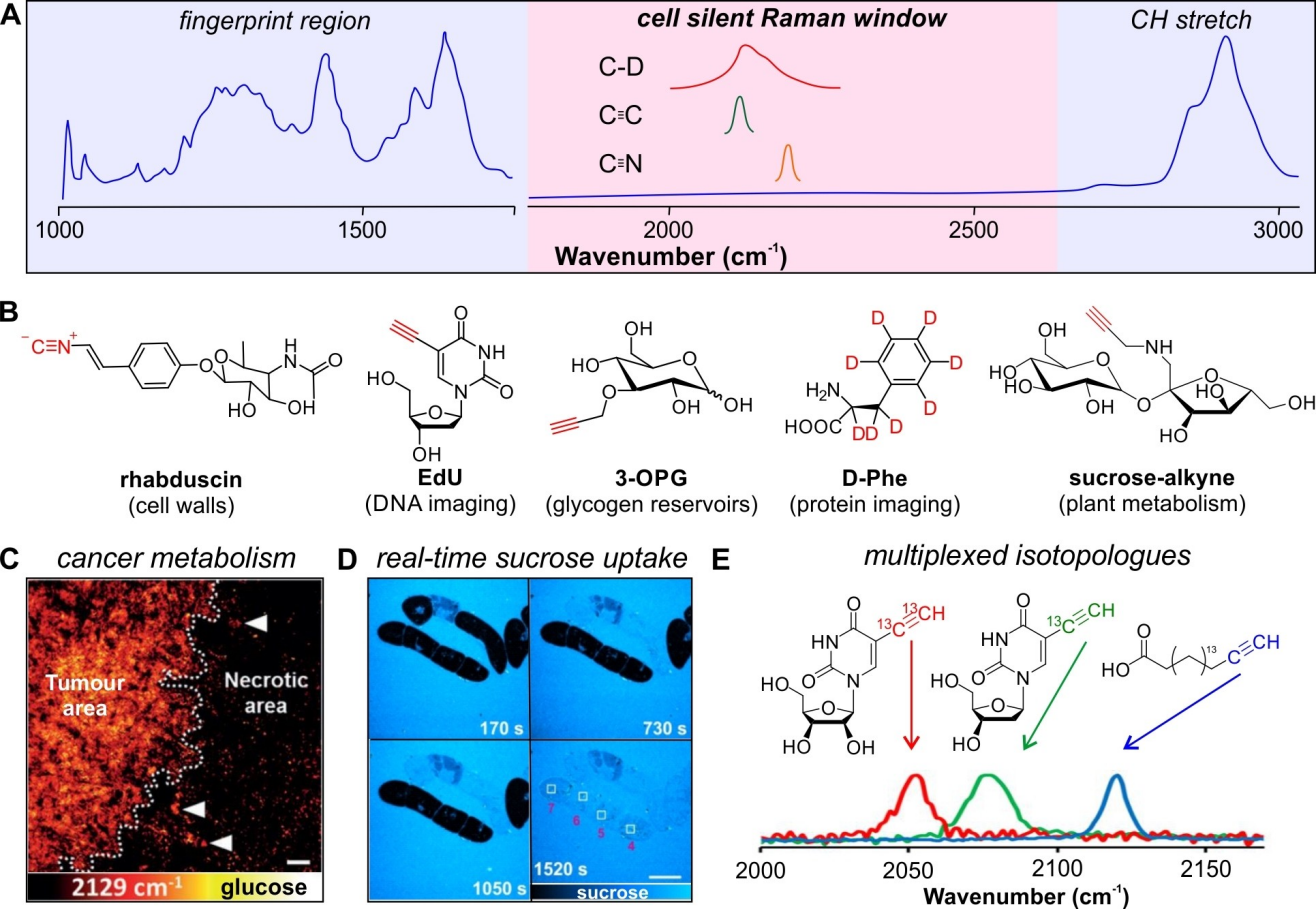
Miniaturized Raman labeling of biometabolites. A) Generic Raman spectrum of mammalian cells highlighting miniaturized Raman labels that produce characteristic peaks in the cell silent region (1800–2600 cm^−1^). B) Examples of vibrational Raman tags (highlighted in red) for imaging small molecule metabolites and examples of biological applications. C) SRS imaging of 3‐OPG in U‐87MG tumor xenograft tissue. The uptake of 3‐OPG is higher in proliferating areas than in the necrotic areas. Reproduced with permission.^[74]^ Copyright 2015 Wiley‐VCH. D) Real‐time uptake of aminopropargyl sucrose into live BY2 cells using SRS microscopy. Reproduced with permission.^[75]^ Copyright 2021 Wiley‐VCH. E) Three‐color multiplex imaging of EU‐^13^C_2_ (RNA), EdU‐^13^C (DNA) and 17‐ODYA (fatty acids) using an alkyne isotopic editing strategy. Reproduced with permission.^[88]^ Copyright 2014 American Chemical Society.

Whereas several Raman microscopy reports have demonstrated label‐free detection of proteins,^[61]^ lipids,^[62]^ DNA^[63]^ and plant cell walls^[64]^ among others, its limited sensitivity and overlapping spectral features have prompted the design of Raman labeling strategies using miniaturized alkynes, nitriles and isotopic tags (Figure 5).

The first example of alkyne tag Raman imaging was demonstrated with the thymidine analogue EdU, a probe that incorporates into nascent DNA to monitor cell proliferation.^[65]^ The alkyne moiety of EdU was detected at 2125 cm^−1^ using spontaneous Raman spectroscopy. Subsequently, stimulated Raman scattering (SRS) microscopy, which offers faster image acquisition rates, has been identified as a versatile imaging modality for biological applications using alkyne probes. For instance, alkyne tag Raman imaging was employed to visualize small molecule drugs,^[66]^ complex natural products^[67]^ and agrochemical agents.^[68]^ With the goal to improve sensitivity, polydiacetylenes showing 10^4^‐fold higher signals compared to single alkyne species were described for rapid visualization of cellular organelles using SRS microscopy.^[69]^ To extend the multiplexing capabilities of alkyne probes, the group of Li generated a multimodal reporter for mitochondrial imaging with SRS—using an internal alkyne group at 2223 cm^−1^—and two‐photon fluorescence via aggregation‐induced emission.^[70]^

Owing to their small size, alkynes and nitriles are interesting minimal groups to track biometabolites. Early examples included the detection of nucleic acids using EdU and EU.^[71]^ Nascent protein synthesis was imaged using homopropargylglycine, a Raman‐active analogue of methionine that is recognised by the cellular translational machinery. Moreover, to demonstrate the application of alkynes in complex biological models, 17‐octadecynoic acid (17‐ODYA) was used to visualize fatty acid metabolism in *C. elegans*.^[72]^ These approaches can also be applied to monosaccharides and polysaccharides. Glucose and sucrose are among the most abundant metabolites in mammals and plants respectively, and larger saccharides make up for a significant part of cellular structural components involved in signalling pathways.^[73]^ For instance, Min and co‐workers reported propargyl tagging to synthesize 3‐O‐propargyl glucose (3‐OPG), which was recognized by native transporters and used to image glucose metabolism in live cells and tissues (Figure 5).^[74]^ Our group synthesized a collection of sucrose analogues, from which aminopropargyl sucrose was used for trafficking studies in live plant cells for the first time (Figure 5).^[75]^ Furthermore, SRS microscopy of sialylated glycans using a similar tagging strategy has been reported.^[76]^ In addition to alkynes, nitrile and isonitrile groups have also been employed as small Raman tags for metabolite detection, such as rhabduscin in bacteria^[77]^ and the small molecule drug, neratinib.^[78]^ Altogether, these studies highlight the biocompatibility of minimally perturbative (<50 Da) alkyne and nitrile tags for metabolite imaging using Raman microscopy.

In parallel to triple bond labels, isotopic substitution strategies are a powerful labeling method due to their extremely small size. Deuterium labeling is attractive because C−D bonds generate Raman shifts within the cell silent region (around 2100 cm^−1^) as demonstrated with the visualization of a wide variety of biomolecules, including proteins,^[79]^ fatty acids,^[80]^ and drugs.^[81]^ Among these, the group of Min reported a general platform referred to as deuterium oxide‐SRS (DO‐SRS) for imaging of de novo lipogenesis in mice.^[82]^ A similar platform termed STRIDE (spectral tracing of deuterium)^[83]^ was reported for the detection of newly synthesized DNA, proteins and glycogen through metabolic incorporation of a deuterated glucose precursor. Similarly, de novo fatty acid synthesis in melanoma cell models was achieved using glucose‐d_7_ as a precursor^[84]^ and, finally, deuterated glutamine^[85]^ and glycogen^[86]^ precursors have been investigated to study the metabolism of cancer cell lines.

Isotopic substitution of carbon has also been described as an alternative methodology for ratiometric and multiplexed detection using SRS microscopy. Proteome turnover in live cells was monitored with ^13^C‐labeled Phe, where the spectral shift from 1004 cm^−1^ (^12^C‐Phe) to 968 cm^−1^ (^13^C‐Phe) enabled ratiometric measurements.^[87]^ A novel strategy for multiplexed SRS imaging was recently reported using ^13^C‐labeled alkynes (Figure 5).^[88]^ In this case, isotopologues of alkynes presented discrete spectral shifts due to the differences in mass of the ^12^C/^12^C, ^13^C/^13^C and ^12^C/^13^C mixed isotopologues, which enabled three‐color detection of DNA, RNA and lipids in live cells. Isotopic editing also enabled ratiometric imaging of cellular glucose metabolism,^[89]^ proving that isotope tags are a powerful means to expand the spectral colors for multiplexed Raman imaging with minimal perturbation to the parent structure.

## 6 Multiplexable and Ratiometric Raman Tags

Multiplexed optical analysis is challenging because of the limited readouts that can be simultaneously detected in complex biological samples. Raman vibrational tags provide transitions with narrow peak widths (≈15 cm^−1^), which in principle allow for the identification of tens of molecular species at the same time.^[90]^

**Figure 6:**
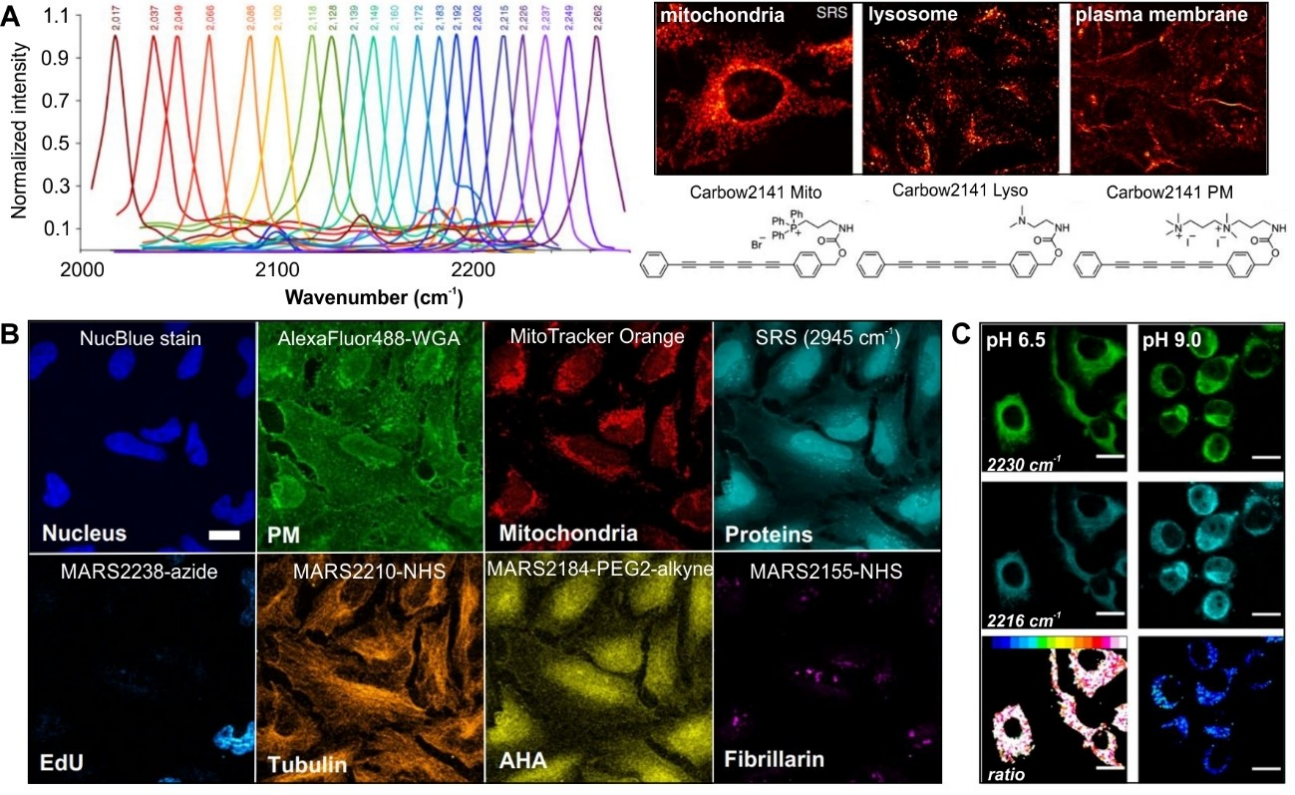
Multiplexed Raman imaging and sensing in mammalian cells. A) Raman spectra of the polyyne “Carbow” collection of multiplexable Raman labels. SRS images of cellular mitochondria, lysosomes and plasma membranes labeled with polyynes containing organelle‐targeting motifs. Reproduced with permission.^[91]^ Copyright 2018 Springer Nature B) 8‐color multiplexed cell imaging using fluorescence and epr‐SRS probes. Fluorescence: NucBlue for nucleus, Alexa‐488‐WGA for plasma membrane (PM), and MitoTracker Orange for mitochondria. SRS: Label‐free detection at 2945 cm^−1^ for proteins, MARS2238‐Azide with EdU for DNA, MARS2210‐NHS labeled antibody for α‐tubulin, MARS2184‐PEG2‐Alkyne with L‐azidohomoalanine (AHA) for nascent proteins and MARS2155‐NHS labeled antibody for fibrillarin. Reproduced with permission.^[96]^ Copyright 2021 Springer Nature under a creative commons license. C) Ratiometric SRS imaging of mitochondrial pH using Mitokyne. SRS images acquired at 2230 cm^−1^ (green, protonated Mitokyne) and 2216 cm^−1^ (cyan, deprotonated Mitokyne). The ratio 2230 cm^−1^/2216 cm^−1^ is featured. Reproduced with permission.^[99]^ Copyright 2021 American Chemical Society under a creative commons license.

The group of Min reported conjugated polyyne scaffolds to detect 20 different targets in live cells (Figure 6).^[91]^ A variety of chemical modifications were used to create mixed isotopologues, including chain elongation (e.g., 2‐yne to 6‐yne), end capping substitutions (e.g., replacement of electron‐donating and electron‐withdrawing groups) and ^13^C isotopic editing. By applying an iterative design strategy, a bespoke family of 20 Raman probes termed “Carbow” (carbon rainbow) was prepared, with each probe exhibiting a discrete spectral frequency within 2017–2280 cm^−1^ for simultaneous imaging of organelle structures and combinatorial barcoding using polymer beads. The strategy was further optimized by incorporating polyyne Raman tags inside polystyrene nanoparticles (termed “Rdots”) for improved sensitivity in the picomolar range.^[92]^ By capitalizing on the high spectral resolution of Raman spectroscopy, multiplexed detection of 14 biomarkers for live cell profiling was reported.^[93]^ In this study, the conjugation of Rdots to antibodies and aptamers was used to analyze cell surface protein abundance, endocytosis activity and metabolic dynamics. Through integration of fluorescence microscopy into a similar SRS platform, Ozeki and co‐workers demonstrated faster visualization of multiplexed samples using an image‐based cytometry approach.^[94]^

An interesting alternative design for multiplexed miniaturized probes was recently reported via coupling of Raman‐active alkyne and nitrile moieties to NIR chromophores.^[90]^ By matching the molecular absorption of the xanthene chromophore to be close to ‐yet not matching the SRS excitation wavelength‐ the authors achieved a dramatic enhancement of the Raman scattering cross‐section (e.g., up to 10^5^‐fold). This process was referred to as electronic pre‐resonance (epr)‐SRS microscopy.^[95]^ The resulting Manhattan Raman scattering (MARS) dyes provided 14 spectrally resolved Raman peaks in the cell silent region and, combined with 4‐channel fluorescence imaging, reached a total of 24 detectable colors in submicromolar concentrations. Further multiplexed capabilities have been achieved by modification of the xanthene core through ring expansion, replacement of the central atoms and isotopic editing of the appended alkyne and nitrile groups (Figure 6).^[96]^

One of the first applications of small molecule ratiometric Raman sensing demonstrated intracellular pH measurement using the pH‐sensitive C≡N stretching frequency of carbonylcyanide **p**‐trifluoromethoxyphenylhydrazone.^[97]^ Building from this concept, the group of Tomkinson synthesized a family of alkyne sensors for intracellular pH sensing, spanning a broad range of pH values, between 2 and 10.^[98]^ A subsequent adaptation of this strategy resulted in the design of Mitokyne as the first small molecule ratiometric pH sensor targeted to mitochondria for SRS microscopy (Figure 6).^[99]^ Briefly, Mitokyne contained a triphenylphosphonium group and a phenolic bisarylbutadiyne, where a red shift of the alkyne band (e.g., 2217 cm^−1^ to 2207 cm^−1^) was gradually observed at increasing pH values.

The ratiometric detection of small cellular messengers including hydrogen sulfide (H_2_S) and ionic species has been possible using Raman‐based techniques. Notably, a ratiometric sensor for H_2_S was reported based on a bisarylbutadiyne probe containing an aromatic azide group that was reduced to aniline in the presence of intracellular H_2_S.^[100]^ Ratiometric Raman sensing of other biologically relevant species (e.g., ions) has also been reported. For instance, the detection of Zn^2+^ in live cells was demonstrated using a chelation‐based Raman probe,^[101]^ whilst fluoride anions were detected through the desilylation of alkyne groups in a paper‐based assay format.^[102]^ The future design of probes that improve the Raman scattering cross‐section of conjugated polyynes will lead to novel chemical structures with additional biological applications for cell‐based studies.

## 7 Activatable Raman Probes for Smart Sensing

**Figure 7:**
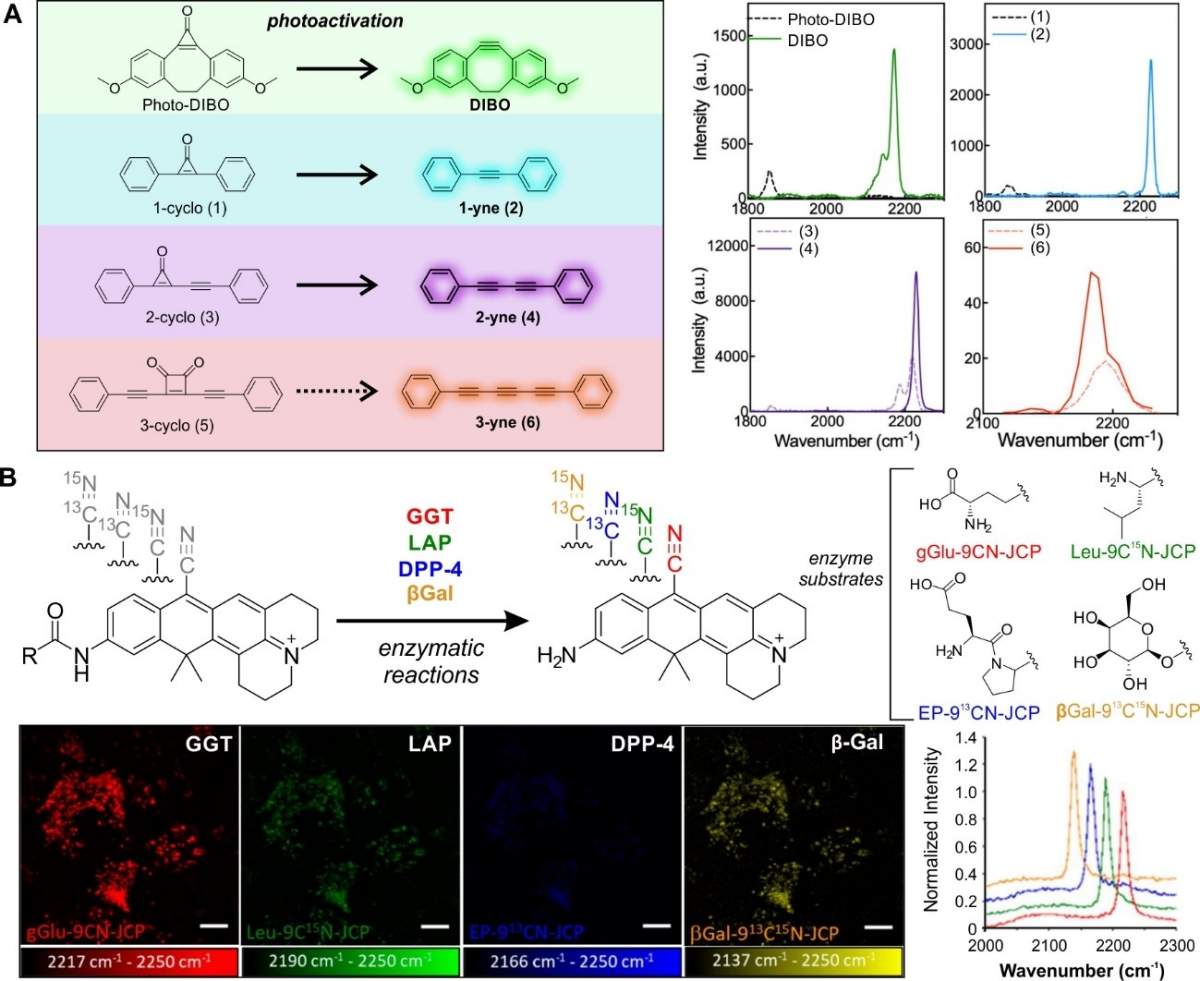
Activatable Raman probes for smart sensing. A) Structures and reactions of alkyne precursors photo‐DIBO, 1‐cyclo, 2‐cyclo and 3‐cyclo as model systems for UV‐activated alkyne generation. Spontaneous Raman and hyperspectral SRS spectra of precursors and products (10 mM). 3‐cyclo (5) did not produce an activatable signal following UV irradiation, and the hyperspectral SRS spectra of (5) and (6) were shown to overlap. Reproduced with permission.^[104]^ Copyright 2022 American Chemical Society. B) Activatable Raman probes based on isotope edited xanthenes for multiple enzyme targets: gGlu‐9CN‐JCP (red) for γ‐glutamyl transpeptidase (GGT), Leu‐9C^15^N‐JCP (green) for leucine aminopeptidase (LAP), EP‐9^13^CN‐JCP (blue) for dipeptidyl peptidase‐4 (DPP‐4) and β‐Gal‐9^13^C^15^N‐JCP (yellow) for β‐galactosidase (βGal). Simultaneous detection of enzyme activities in live A549 cells in epr‐SRS images (where the background image at 2250 cm^−1^ was subtracted in each case), together with the normalized SRS spectra of all 4 probes. Reproduced with permission^[108]^ under ACS Editors’ Choice. Copyright 2020 American Chemical Society.

Activatable probes have found widespread application in imaging and sensing with the real‐time analysis of multiple biomolecules including enzymes, ionic species and cell receptors.^[103]^ Similar to the fluorescent probes that switch from off to on states, parallel strategies have been developed for Raman reporters. Du and Wei developed a cyclopropenone caging system for photoactivatable SRS microscopy^[104]^ relying on the photoconversion of cyclopropenones to alkynes, which in turn created a turn‐on response in the cell silent region of the Raman spectrum (Figure 7). Initial studies showed that the compound Photo‐DIBO presented weak Raman scattering at 1851 cm^−1^, which, after UV irradiation, displayed an intense characteristic alkyne peak at 2171 cm^−1^. This methodology was extended to create polyyne structures with organelle‐targeting motifs for site‐specific intracellular activation, and isotopic editing of the cyclopropenone ring structure for extended multiplexable capabilities. To improve the detection sensitivity of photoactivatable Raman probes, Wei and co‐workers have recently investigated the potential application of this methodology using epr‐SRS.^[105]^

The narrow peaks of Raman tags represent an advantage over fluorescent structures for multicolor photoswitchable imaging. For instance, photoswitchable SRS imaging of cis‐1,2‐dicyano‐1,2‐bis(2,4,5‐trimethyl‐3‐thienyl)ethene was reported, where its two isomers (i.e., an open form and a closed form) were interchangeable by irradiation with UV or visible light, respectively.^[106]^ Notably, light activatable imaging was demonstrated in HeLa cells, highlighting the biocompatibility of this approach. Another early example of photoswitching SRS microscopy was demonstrated using alkyne diarylethenes (DTEs).^[107]^ The alkyne group shifted reversibly upon photoisomerization of the conjugated DTE when irradiated by UV or visible light to yield on or off states, respectively. After UV irradiation, the photoisomerization red‐shifted its absorption spectrum and induced a frequency shift of the conjugated alkyne groups (2194 cm^−1^ to 2214 cm^−1^) and an intensity increase via epr‐SRS. Photoswitchable SRS imaging was achieved at 2194 cm^−1^ (DTE‐Ph‐Mito) in live cells through the incorporation of a pyridinium group for mitochondrial targeting, whilst multicolor SRS imaging using other Raman probes (e.g., EdU) was demonstrated.

Ozeki, Kamiya and co‐workers reported some of the first activatable Raman probes for multiplexed enzyme detection (Figure 7).^[108]^ Enzyme‐activatable probes were based on the 9‐cyanopyronine scaffold containing an enzyme recognition motif and a nitrile group for bioorthogonal Raman sensing. To enable multiplexed detection of mixed enzyme targets, the authors employed an isotopic editing strategy of the nitrile group using ^12^C/^13^C and ^14^N/^15^N. The authors simultaneously detected 4 targeted enzymes in live A549 cells, highlighting the potential of this technique to visualize protein heterogeneity in complex samples. In an alternative strategy, chemical activation of epoxy ketones to yield a terminal alkyne via Eschenmoser–Tanabe fragmentation using p‐toluenesulfonyl hydrazine was reported.^[109]^ The activation of a steroid analogue probe was imaged using SRS microscopy at 2115 cm^−1^. Altogether, these studies demonstrate that activatable Raman probes offer a unique approach for multiplexed imaging with potential applications to investigate the complex interplay of biological systems.

## 8 Conclusions

In parallel to label‐free imaging, the miniaturization of chemical labels has become an integral challenge in optical probe development (Table 1). The fine tuning of fluorophores as small as a single aryl‐substituted moiety has rendered bright fluorescent structures for non‐perturbative derivatization of biomolecules, including environmentally sensitive dyes, fluorescent amino acids and nucleobases. Fluorophores are easy to implement in biological assays, yet their bulkiness can impair some biological functions. Alongside the development of small sized fluorophores, chemists have described new approaches to introduce minimal Raman labels into biomolecules. Raman‐active labels are advantageous in that they can be 1) as small as a single isotope, and 2) engineered to detect several targets simultaneously. The chemistry of Raman tagging has advanced very rapidly in the last decade and produced novel analogues for metabolic studies and multiplexed detection of dozens of biomarkers. Altogether, the adaptation of these and future optically active miniaturized labels will create multiple avenues to interrogate biological systems in the least invasive manner possible.

**Table 1:** Representative optical probes including miniaturized tags for fluorescence and Raman imaging.

Compound	Structure	Tag type	Reporter	Optical modality	ΔM_W_ fold increase	Ref.
3‐OPG	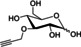	Metabolites	Propargyl	Raman	1.21	[74]
C_3_NBD ceramide	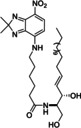	Metabolites	NBD fluorophore	Fluorescence	1.49	[15a]
Sucrose‐alkyne	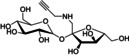	Metabolites	Propargyl	Raman	1.11	[75]
dCmBdp	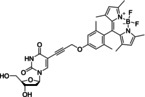	Nucleic acids	BODIPY fluorophore	Fluorescence	2.78	[33a]
dioxT	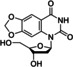	Nucleic acids	Benzopyrimidine	Fluorescence	1.33	[30]
Thienoguanosine	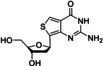	Nucleic acids	Thienopyrimidine	Fluorescence	1.06	[28]
EdU	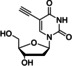	Nucleic acids	Alkyne	Raman	1.11	[65]
Pyrazolo‐quinazolines	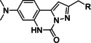	Amino acids	Pyrazolo‐quinazoline	Fluorescence	3.05	[54]
Trp‐BODIPY	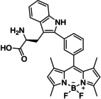	Amino acids	BODIPY fluorophore	Fluorescence	2.58	[40]
L‐Phe(d8)	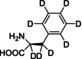	Amino acids	Deuterium atoms	Raman	1.05	[79]
Rhabduscin	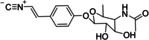	Organelle tags	Isonitrile	Raman	1	[77]
mitoSBF	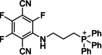	Organelle tags	Modified benzene	Fluorescence	n.a.	[23]
Carbow2141 Mito	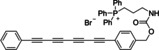	Multiplexing	Conjugated alkynes	Raman	n.a.	[91]
MARS2210‐NHS	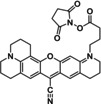	Multiplexing	Nitrile	Raman	n.a.	[96]
CN‐JCP	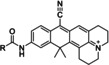	Activatable	Nitrile	Raman	n.a.	[108]
Photo‐DIBO	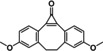	Activatable	Cyclopropenone	Raman	n.a.	[104]

## Conflict of interest

The authors declare no conflict of interest.

## Biographical Information

*Sam Benson received an MSc in Medicinal and Biological Chemistry from the University of Nottingham in 2016. Sam carried out his PhD under the supervision of Prof Marc Vendrell at the University of Edinburgh developing novel theranostics for cancer, including the synthesis of small fluorophores and photosensitizers. He has subsequently remained in the same group as a postdoctoral research associate, where his work focuses on the development of new systems for photodynamic therapy of glioblastoma*.

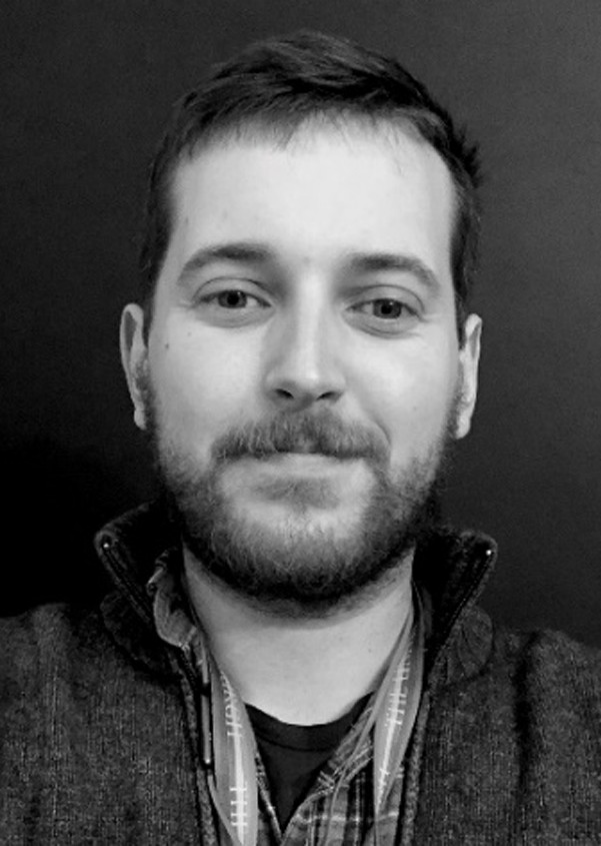

## Biographical Information

*Fabio de Moliner obtained his PhD in Organic Chemistry from the University of Genova in 2010 under the guidance of Prof Andrea Basso. After graduation, he joined the research group of Prof Christopher Hulme at the University of Arizona (Tucson) as a postdoctoral research assistant to focus on the design and application of chemical methods based on multicomponent and domino processes. He joined the Vendrell group at the University of Edinburgh in 2015. His main research interests are the development of optical probes for* in vivo *imaging with a focus on the preparation of fluorescent amino acids and the labeling of small metabolites*.

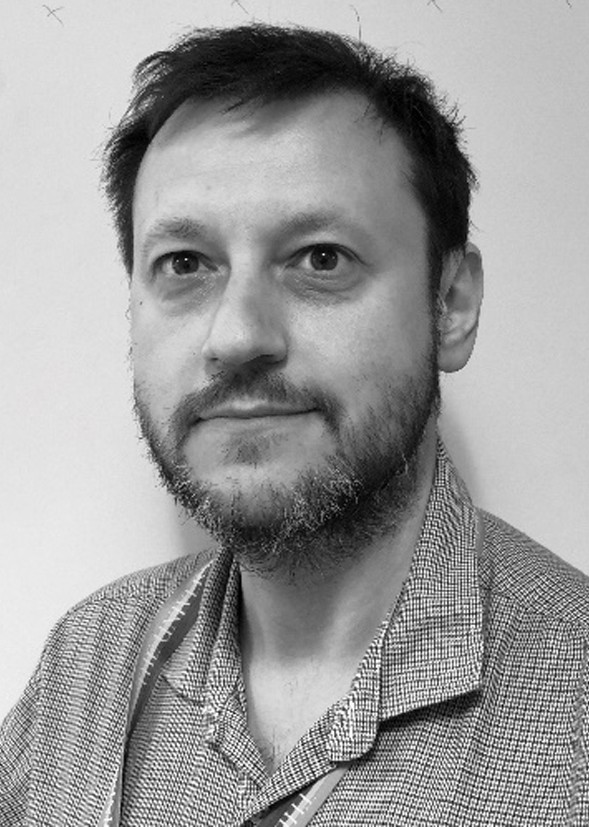

## Biographical Information

*William Tipping obtained his PhD in Chemistry from the University of Edinburgh in 2017 under the guidance of Prof Alison Hulme and Prof Valerie Brunton. After a brief postdoctoral position in the same group, he joined the University of Strathclyde in 2019 to work with Prof Duncan Graham and Prof Karen Faulds. His research interests include the development of imaging probes for Raman microscopy, and the development of stimulated Raman scattering microscopy for biomedical imaging applications*.

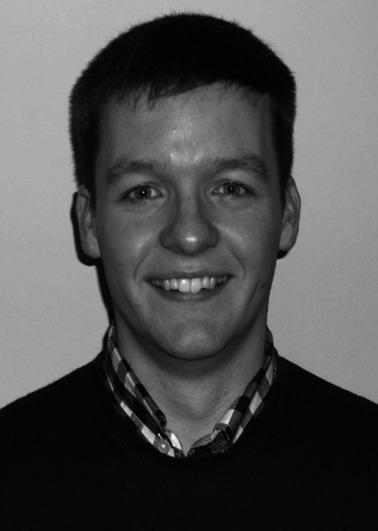

## Biographical Information

*Marc Vendrell graduated in Chemistry at the University of Barcelona in 2007. He then joined the Singapore Bioimaging Consortium to work in synthetic fluorophores for optical imaging. In 2012 he started his independent career at the University of Edinburgh to develop optical probes for imaging immune function in humans. His research has led to the commercialization of several fluorescent probes and to the use of different molecular reagents for first‐in‐human imaging studies, which has been recognized internationally with several awards and distinctions. Since 2020 he is appointed as Chair of Translational Chemistry and Biomedical Imaging at the College of Medicine in Edinburgh*.